# Pro-inflammatory properties of stromal cell-derived factor-1 (CXCL12) in collagen-induced arthritis

**DOI:** 10.1186/ar1806

**Published:** 2005-08-25

**Authors:** Bert De Klerck, Lies Geboes, Sigrid Hatse, Hilde Kelchtermans, Yves Meyvis, Kurt Vermeire, Gary Bridger, Alfons Billiau, Dominique Schols, Patrick Matthys

**Affiliations:** 1 Laboratory of Immunobiology, Rega Institute, Katholieke Universiteit Leuven, Leuven, Belgium; 2 Laboratory of Virology and Chemotherapy, Rega Institute, Katholieke Universiteit Leuven, Leuven, Belgium; 3 AnorMED, Langley, British Columbia, Canada

## Abstract

CXCL12 (stromal cell-derived factor 1) is a unique biological ligand for the chemokine receptor CXCR4. We previously reported that treatment with a specific CXCR4 antagonist, AMD3100, exerts a beneficial effect on the development of collagen-induced arthritis (CIA) in the highly susceptible IFN-γ receptor-deficient (IFN-γR KO) mouse. We concluded that CXCL12 plays a central role in the pathogenesis of CIA in IFN-γR KO mice by promoting delayed type hypersensitivity against the auto-antigen and by interfering with chemotaxis of CXCR4^+ ^cells to the inflamed joints. Here, we investigated whether AMD3100 can likewise inhibit CIA in wild-type mice and analysed the underlying mechanism. Parenteral treatment with the drug at the time of onset of arthritis reduced disease incidence and modestly inhibited severity in affected mice. This beneficial effect was associated with reduced serum concentrations of IL-6. AMD3100 did not affect anti-collagen type II antibodies and, in contrast with its action in IFN-γR KO mice, did not inhibit the delayed type hypersensitivity response against collagen type II, suggesting that the beneficial effect cannot be explained by inhibition of humoral or cellular autoimmune responses. AMD3100 inhibited the *in vitro *chemotactic effect of CXCL12 on splenocytes, as well as *in vivo *leukocyte infiltration in CXCL12-containing subcutaneous air pouches. We also demonstrate that, in addition to its effect on cell infiltration, CXCL12 potentiates receptor activator of NF-κB ligand-induced osteoclast differentiation from splenocytes and increases the calcium phosphate-resorbing capacity of these osteoclasts, both processes being potently counteracted by AMD3100. Our observations indicate that CXCL12 acts as a pro-inflammatory factor in the pathogenesis of autoimmune arthritis by attracting inflammatory cells to joints and by stimulating the differentiation and activation of osteoclasts.

## Introduction

Among chemokines, CXCL12 (formerly stromal cell-derived factor 1) is unique in that it binds to one single chemokine receptor, CXCR4, which itself is recognized by no other chemokines [1-3]. CXCL12 is produced physiologically in various tissues and its receptor CXCR4 is also expressed on various haematopoietic and non-haematopoietic cells. By binding to heparan sulphate proteoglycans, secreted CXCL12 can adhere to certain cells such as bone marrow stromal cells. Through this mechanism, CXCL12-CXCR4 interaction plays an important role in homing of myeloid and lymphoid cells to specific sites in bone marrow or secondary lymphoid organs. CXCR4 also acts as an important co-receptor for HIV entry into CD4^+ ^human lymphocytes [4]. Like other members of the chemokine family, CXCL12 may play a role in inflammatory diseases. Specifically, there is increasing evidence that CXCL12 plays a crucial role in patients with rheumatoid arthritis (RA). In RA patients, abnormally high concentrations of CXCL12 in synovial fluid and overexpression of CXCL12 in synovial cells have been found [5-8]. Moreover, CXCR4^+ ^leukocytes in synovia were found to be significantly more abundant [7]. Evidence also points to a role for CXCL12 in positioning CXCR4^+ ^T and B cells to distinct synovial microdomains as well as in retaining these cells within the inflamed synovial tissue [9]. CXCL12 induces migration of monocytes into human arthritic synovium transplanted into severe combined immunodeficiency (SCID) mice [10]. In addition to exerting these effects on cell migration, CXCL12 also induces angiogenesis during RA development [8] and stimulates chondrocytes to release matrix metalloprotease 3 (MMP3), a matrix-degrading enzyme involved in cartilage destruction [5].

Availability of specific inhibitors of the CXCL12-CXCR4 interaction has allowed the demonstration of the involvement of CXCL12 in experimental animal diseases. One such inhibitor is the bicyclam drug AMD3100, originally discovered as an anti-HIV compound and which specifically interacts with CXCR4 [11,12]. We found that AMD3100 reduces the severity of collagen-induced arthritis (CIA) in mice, a model for RA in man. The study was done on IFN-γ knock-out (IFN-γR KO) DBA/1 mice, which are more susceptible to CIA than wild-type mice [13]. Reduced severity of arthritis was associated with a significant reduction in the delayed type of hypersensitivity (DTH) response to the auto-antigen collagen type II (CII). The majority of leukocytes harvested from inflamed joints of arthritic IFN-γR KO mice were found to be CD11b^+^, and AMD3100 was demonstrated to interfere with the chemotaxis induced *in vitro *by CXCL12 on purified CD11b^+ ^splenocytes. We concluded that CXCL12 contributes to the pathogenesis of CIA in these mutant mice by promoting DTH and by interfering with migration of CD11b^+ ^cells into joint tissues.

A major difference in the pathogenesis of CIA between IFN-γR KO and wild-type mice is the presence of more extensive extramedullary myelopoiesis in IFN-γR KO mice, leading to an expansion of CD11b^+ ^cells that can act as DTH and arthritogenic effectors [14-16]. Thus, in IFN-γR KO mice, the balance between cellular (DTH) and humoral autoimmune responses seems to be shifted towards DTH, and this bias may in part explain the beneficial effects of AMD3100 in IFN-γR KO mice. We have tested this hypothesis in the present study. We investigated to what extent AMD3100 affects CIA in wild-type mice and, if so, which mechanisms are involved. We found that AMD3100 does inhibit the disease but that, in contrast to IFN-γR KO mice, this was not associated with reduction in DTH reactivity against CII. We show that, aside from inhibiting chemotaxis *in vitro*, AMD3100 also inhibits the CXCL12-elicited cell migration into subcutaneous air pouches *in vivo*. In addition, we found CXCL12 to be able to enhance receptor activator of NF-κB ligand (RANKL)-induced osteoclast differentiation from splenocytes and to increase osteoclast activity, two effects that were counteracted by AMD3100.

## Materials and methods

### Induction of collagen-induced arthritis

Mice of the DBA/1 strain were bred in the Experimental Animal Centre of the Katholieke Universiteit Leuven (Leuven, Belgium). The experiments were performed in 8- to 12-week-old male mice that were age-matched within each experiment.

CII from chicken sternal cartilage (Sigma-Aldrich Co., St Louis, MO, USA) was dissolved at 2 mg/ml in PBS containing 0.1 M acetic acid by stirring overnight at 6°C. The CII solution was emulsified with an equal volume of complete Freund's adjuvant (CFA; Difco Laboratories, Detroit, MI, USA) with added heat-killed *Mycobacterium butyricum *(Difco), reaching a final *Mycobacterium* content of 750 μg/ml emulsion. Mice were injected intradermally with 100 μl emulsion at the base of the tail on day 0.

Mice were examined daily for signs of arthritis. The disease severity was recorded for each limb, as described in [17]: score 0, normal; score 1, redness and/or swelling in one joint; score 2, redness and/or swelling in more than one joint; score 3, redness and/or swelling in the entire paw; score 4, deformity and/or ankylosis.

All animal experiments were approved by the local ethical committee (University of Leuven).

### Treatment with AMD3100

AMD3100 was provided by AnorMED (Langley, British Columbia, Canada). For the treatment with AMD3100, Alzet osmotic minipumps model 2002 (DURECT corporation, Cupertino, CA, USA) were subcutaneously implanted at the dorsolateral part of the body. During the procedure, the mice were anaesthetized with a solution of PBS containing 0.2% (v/v) Rompun (Bayer, Brussels, Belgium) and 1% (v/v) Ketalar (Parke-Davis, Zaventem, Belgium). The minipumps delivered AMD3100 at a constant rate of 600 μg/day for 14 days.

### Histology

Fore and hind limbs (ankles and interphalanges) were fixed in 10% formalin and decalcified with formic acid. Paraffin sections were haematoxylin stained. Severity of arthritis was evaluated blindly using three parameters: infiltration of mono- and polymorphonuclear cells; hyperplasia of the synovium; and bone destruction. Each parameter was scored on a scale from 0 to 3: score 0, absent; score 1, weak; score 2, moderate; score 3, severe.

### Serum anti-collagen type II ELISA

Individual sera were tested for the amount of anti-CII antibody by ELISA, as described previously [17]. Briefly, ELISA plates (Maxisorp, Nunc, Wiesenbaden, Germany) were coated overnight with chicken CII (1μg/ml; 100 μl/well; Sigma-Aldrich Co, St Louis, MO, USA) in coating buffer (50 mM Tris-HCL, pH 8.5; 0.154 mM NaCl) followed by a 2 h incubation with blocking buffer (50 mM Tris-HCl, pH 7.4; 154 mM NaCl and 0.1% (w/v) casein). Serial twofold dilutions of the sera and the standard were incubated overnight in assay buffer (50 mM Tris-HCl; pH 7.4; 154 mM NaCl and 0.5% Tween-20). The quantification of total IgG was done by ELISA making use of a standard with known IgG concentration. For determination of the IgG2a, IgG2b and IgG1 antibody concentrations, a standard of arbitrary U/ml was used (standard = 1,000 U/ml). Plates were then incubated for 2 h with biotinylated rat antibody to mouse total IgG, IgG2a, IgG2b or IgG1 (Zymed Laboratories, San Francisco, CA, USA). Plates were washed and incubated for 1 h with streptavidin-peroxidase. Finally, the substrate 3,3'-5,5'-tetramethyl-benzidine (Sigma-Aldrich Co.) in reaction buffer (100 mM sodium acetate/citric acid, pH 4.9) was added. Reaction was stopped using 50 μl H_2_SO_4 _2 M and absorbance was determined at 450 nm.

### Delayed-type hypersensitivity experiments

For evaluation of DTH reactivity, CII/CFA-immunized mice were subcutaneously injected with 10 μg of CII/20 μl PBS in the right ear and with 20 μl PBS in the left ear. DTH response was calculated as the percentage swelling (the difference between the increase of thickness of the right and the left ear, divided by the thickness of the ear before challenge, multiplied by 100).

### Assays for *in vivo *leukocyte migration and for *in vitro *chemotaxis

For the *in vivo *assay, mice were treated with AMD3100 or PBS as described above. The assay was performed on the last day of the treatment. Six days before, mice were subcutaneously injected at the dorsolateral site of the body with 2.5 ml of sterile air, creating a subcutaneous air pouch. At day three before the assay, injection with 2.5 ml sterile air was repeated at the same location. The chemotactic assay was performed by injecting 1 ml 0.9% (w/v) NaCl/CXCL12 2 μg or 0.9% (w/v) NaCl alone into the air pouch (human CXCL12 was provided by Dr I Clark-Lewis, University of British Columbia, Vancouver, BC, Canada). Two hours later, cells were washed out of the air pouch by 2 ml PBS/FCS 2% (v/v) and cells were immediately counted with a light microscope in the Burker chamber.

*In vitro *chemotactic assays were performed at day 21 post immunization. Spleens were isolated and passed through cell strainers to obtain a single cell suspension. Erythrocytes were removed by lysis with NH_4_Cl (0.83% (w/v) in 0.01 M Tris-HCl, pH 7.2; two consecutive incubations of 5 and 3 min, 37°C). Splenocytes of three mice were pooled and incubated with AMD3100 at different concentrations in assay buffer (HBSS, 20 mM Hepes, 0.2% (w/v) BSA, pH 7.2). Transwell filter membranes (5 μm pore; Costar, Boston, MA, USA) were placed in the wells of a 24-well plate, each containing 600 μl buffer with or without CXCL12 at a concentration of 100 ng/ml (human CXCL12 was provided by Dr I Clark-Lewis). 10^6 ^cells were loaded on each Transwell filter. The plate was then incubated for 3.5 h at 37°C, whereupon the filter inserts were carefully removed. The migrated cells were collected and counted in a flow cytometer (FACScalibur; Becton Dickinson, San Jose, CA, USA) as described [18-20]. The number of cells is represented as the number of counts registered during a two-minute acquisition (number of cells/2 minutes).

Migrated cells were incubated with anti-CD16/CD32 Fc-blocking antibodies (BD Biosciences Pharmingen, San Diego, CA, USA) and washed with PBS. After washing, the cells were stained for 30 minutes with anti-CD4-PE, anti-CD8-FITC, anti-CD19-PE or anti-CD11b-FITC (BD Biosciences Pharmingen). Cells were washed, fixed with 0.37% formaldehyde in PBS, and analysed by a FACScalibur flow cytometer (Becton Dickinson).

The chemotactic index was calculated as the number of migrated cells obtained with 100 ng/ml CXCL12 divided by the number of cells in the negative control without CXCL12.

### Flow cytometric analysis of cells from joint cavities

Cells from joint cavities were obtained by inserting a 25-gauge needle into the ankle joint. Cold PBS (800 μl) was injected into the joint cavity. Fluid exiting spontaneously from the opening was collected and was only used when it was found to contain <5% of erythrocytes. Cells were washed and resuspended in cold PBS. Cells were incubated with anti-CD16/anti-CD32 Fc-receptor-blocking antibodies (BD Biosciences Pharmingen). After washing, the cells were stained for 30 minutes with anti-CD11b-FITC and anti-CXCR4-PE or isotype control rat IgG2b (BD Biosciences Pharmingen). Cells were washed, fixed with 0.37% formaldehyde in PBS, and analysed by a FACScalibur flow cytometer (Becton Dickinson).

### Polymerase chain reaction

Synovial tissues from the ankle joints were carefully isolated under a stereomicroscope. Total RNA was extracted with Trizol reagent (Invitrogen, Paisley, Scotland, UK), in accordance with the manufacturer's instructions. cDNA was obtained by reverse transcription with a commercially available kit (Thermoscript; Invitrogen) with oligo(dT)_20 _as primer.

For PCR reactions we used a TaqMan^® ^Assays-on-Demand™ Gene Expression Product from Applied Biosystems (Foster City, CA, USA; assay ID Mm00445552_m1). Expression levels of the gene were normalized for 18S RNA expression.

### Cytokine detection in serum and cultured medium

Control-treated and AMD3100-treated mice were bled both before and 6 h after intraperitoneal injection with 10 μg anti-CD3. Sera were collected and pooled. This allowed us to determine the concentrations of the following cytokines: IL-1β, IL-2, IL-4, IL-6, IL-10, IL-12, tumour necrosis factor-α and IFN-γ.

Spleens of three mice were isolated on day 21 after immunization and were passed through cell strainers to obtain a single cell suspension. Erythrocytes were removed by lysis with NH_4_Cl (0.83% (w/v) in 0.01 M Tris-HCl, pH 7.2; two consecutive incubations of 5 and 3 minutes, 37°C). Splenocytes of the mice were pooled and cultured in a 96-well plate. 10^5 ^cells were cultured in one well in Roswell Park Memorial Institute (RPMI) medium alone, RPMI with mouse CXCL12 (0.1 μg/ml) (PeproTech, London, UK), or RPMI with mouse CXCL12 and AMD3100 (25 μg/ml). Supernatant was collected after 48 h. Detection of cytokine concentrations in serum and cultured medium was done with the Endogen SearchLight™ array (Pierce Boston Technology, Woburn, MA, USA).

### *In vitro *induction of osteoclast formation by splenocytes

Spleens were isolated on day 21 after immunization and were passed through cell strainers to obtain a single cell suspension. Erythrocytes were removed by lysis with NH_4_Cl (0.83% (w/v) in 0.01 M Tris-HCl, pH 7.2; two consecutive incubations of 5 and 3 minutes, 37°C). Leukocytes from the blood were obtained by lysis of red blood cells by two incubations (5 and 3 minutes at 37°C) with NH_4_Cl solution (0.083% (w/v) in 0.01 M Tris-HCl; pH 7.2). Remaining cells were washed two times with ice-cold PBS.

Splenocytes were suspended in Minimal Essential Medium alpha Medium (α-MEM) containing 10% (v/v) FCS (GIBCO, Invitrogen corporation, Paisley, Scotland, UK). Cells (2.5 × 10^5^) in a total volume of 400 μl were seeded in chamber slides (LAB-TEK Brand Products, Nalge Nunc International, Naperville, IL, USA). Cells were incubated with macrophage colony stimulating factor (M-CSF; 20 ng/ml) + CXCL12 (0.1 or 0.5 μg/ml; AnorMED), with M-CSF + RANKL (100 ng/ml) + CXCL12 or with M-CSF + RANKL + CXCL12 + AMD3100 (25 μg/ml; AnorMED). M-CSF and RANKL were obtained from R&D Systems Europe (Abingdon, UK). On day 4, supernatants were removed and cultures were provided with fresh media and stimuli. On day 7, media were removed and cells were stained for the presence of tartrate-resistant acid phosphatase (TRAP) (described below).

### Pit-forming assay

Splenocyte suspensions were obtained as described above and resuspended in α-MEM containing 10% (v/v) FCS (GIBCO, Invitrogen Corporation). 10^6 ^cells were cultured for 5 days with M-CSF (20 ng/ml) and RANKL (100 ng/ml), both from R&D systems Europe, on transparent quartz slides coated with a calcium phosphate film (BioCoat Osteologic Discs; BD Biosciences Pharmingen). On day 6, media were removed and replaced with media containing M-CSF, M-CSF + CXCL12 (0.5 μg/ml), M-CSF + CXCL12 + AMD3100 (25 μg/ml) or M-CSF + AMD3100. Another two days later, cells were removed and resorption pits were quantified using a Leitz DM RBE microscope equipped with a colour video camera (Optronics Engineering, Goleta, CA, USA) and attached to a computer-aided image analysing system (Bioquant, R&M Biometrics, Nashville, TN, USA). Quantification and size determination of the pits was performed at a magnification of ×20 in 15 areas of constant size, positioned adjacent to one another and spanning the whole quartz slide. In all slides, the minimum threshold of a pit surface area was set to 50 μm^2^. Upon thresholding, the number and square surface of plaques are determined automatically.

## Results

### Inhibition of collagen-induced arthritis by AMD3100 in DBA/1 wild-type mice

**Figure 1:**
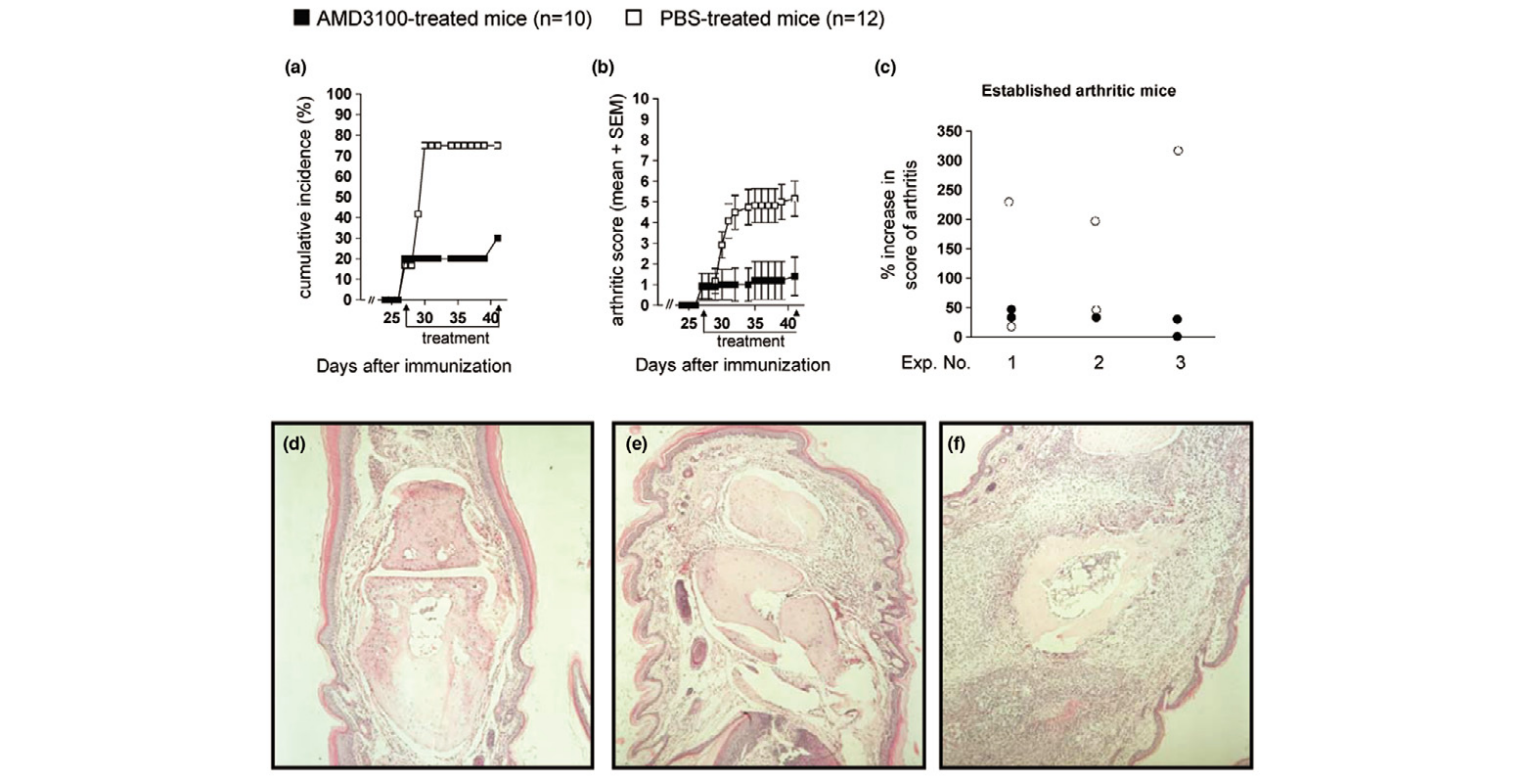
Inhibition of collagen-induced arthritis in DBA/1 mice by treatment with AMD3100. Mice were immunized on day 0 with collagen type II in complete Freund's adjuvant and were observed for symptoms of arthritis. On day 27, when the first symptoms of arthritis appeared, the mice were divided into two groups in a way that a similar incidence and a similar average clinical score was reached in both groups. On this day, mice of one group were implanted with osmotic minipumps, delivering AMD3100 for two weeks at a constant rate of 600 μg/day. Mice of the other group were implanted with pumps containing PBS. The **(a) **cumulative incidence and **(b) **mean arthritic score ± standard error of the mean (SEM) for AMD3100-treated and control-treated mice are shown. Average group scores of arthritis were significantly different from day 30 onwards (p ≤ 0.05 on day 30; p ≤ 0.01 from day 31 till the end of the experiment, Mann-Whitney U test). **(c) **Evaluation of disease progression in mice with established arthritis at initiation of treatment with AMD3100. Circles represent percentage increase in scores of arthritis for individual mice at the end of the treatment. Data show the results of three individual experiments (explained in more detail in the legend of Table 1). **(d-f) **Histological analysis of the joints. On the last day of treatment, five mice out of both groups with a mean score representing the average group score, were selected and sacrificed. Paraffin sections of the fore and hind limbs were haematoxylin stained and histological examination was performed. Representative pictures are shown. **(d) **Joint of an AMD3100-treated mouse without clinical symptoms showing normal histological appearance. **(e) **Joints of arthritic AMD3100-treated mice show a weak infiltration of mono- and polymorphonuclear cells and hyperplasia of the synovium. **(f) **Joint section of a PBS-treated mouse, showing moderate to severe infiltration of leukocytes, hyperplasia and bone destruction.

In a first experiment, DBA/1 mice were immunized with CII in CFA. The symptoms of arthritis started to appear on day 27; 4 mice out of 22 showed redness and/or swelling in one of their joints. On that day, mice were divided in two subgroups, matched for incidence and average clinical score. In one group, mice were implanted with osmotic minipumps releasing AMD3100 at a constant rate of 600 μg/day. Mice in the other group were implanted with pumps delivering PBS. From previous experience and according to the manufacturer's specification sheet, the minipumps are known to be active for two weeks. Mice were scored six times a week for symptoms of arthritis. Cumulative incidence and mean scores of arthritis in both groups during the experiment are shown in Fig. 1a,b. The cumulative incidence of arthritis rapidly increased in the control mice, but remained stable in the AMD3100-treated animals (Fig. 1a). In fact, after initiation of treatment, 7 out of 10 mice in the PBS group developed arthritis within 3 days, whereas in the AMD3100-treated group only a single mouse out of 8 developed symptoms 13 days after initiation of the treatment. Correspondingly, the mean arthritic group score gradually increased in controls, but not in AMD3100-treated animals (Fig. 1b). The beneficial effect of AMD3100 in CIA was confirmed in two additional experiments. The data of the three experiments are summarized in Table 1: during the treatment, in total only 2 out of 20 AMD3100-treated mice developed signs of arthritis against 16 out of 22 controls.

Considering all arthritic mice, the average clinical score of arthritic mice was lower in the treated mice, although the difference compared with that in the arthritic control mice was not statistically significant. Thus, the significantly lower average scores reached in the AMD3100-treated group, when all mice are considered, reflects mainly the lower incidence in this group. In addition, evaluation of disease progression in each of the individual mice having arthritis signs at the initiation of treatment revealed lower percent increases in disease scores in the AMD3100- than in the PBS-treated group (Fig. 1c), suggesting that AMD3100 can also exert a beneficial effect on evolving arthritis. These *in vivo *results show that AMD3100 treatment of CIA initiated at first appearance of symptoms is effective against the development and progression of the disease.

### Reduced histological symptoms of arthritis in AMD3100-treated mice

To ascertain that the protective effect of AMD3100 with respect to the clinical symptoms of arthritis was also manifest at the histological level, five mice from each group (Table 1, experiment 1) were sacrificed for histological examination of the joints. These mice were selected such that their mean clinical scores corresponded to the average score of the entire group.

Haematoxylin-stained sections showed that the absence of redness and swelling in AMD3100-treated mice corresponded with the absence of infiltration of immunocompetent cells and tissue destruction (Fig. 1d). Histological examination of joint sections of AMD3100-treated mice that did show clinical symptoms of arthritis revealed a weak hyperplasia and infiltration of mono- and polymorphonuclear cells in the synovium (Fig. 1e). Sections of arthritic PBS-treated mice showed a moderate to severe infiltration, hyperplasia of the synovium and bone destruction (Fig. 1f).

### AMD3100 does not interfere with humoral or cellular responses to collagen type II

The pathogenesis of CIA is generally considered to depend on both humoral and cellular immunity against CII. To see whether inhibition of CIA by AMD3100 acts via modulation of either of these, we measured specific anti-CII antibodies and DTH reactivity against CII. These tests were performed on day 14 after implantation of minipumps.

**Figure 2:**
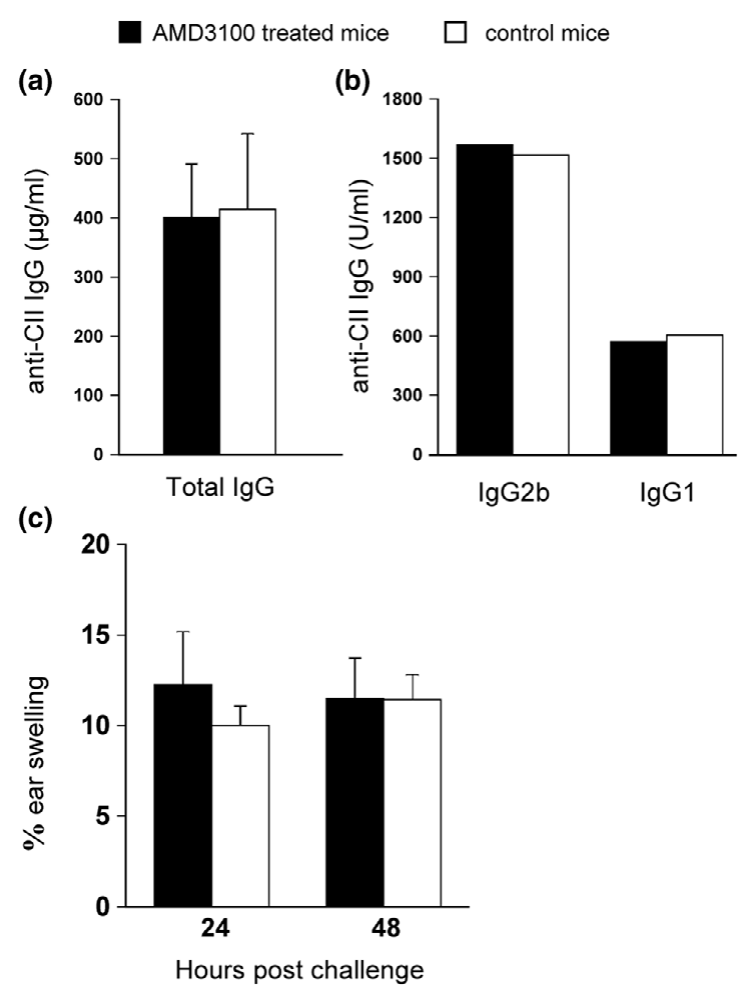
AMD3100 in wild-type mice does not interfere with the humoral or the cellular response. At the end of the two week treatment (day 41), blood was collected from five mice out of each group. **(a) **Sera of individual mice were analyzed for total anti-CII IgG, using an absolute standard. Bars represent averages plus standard error of the mean of five mice. **(b) **Equal quantities of the sera were pooled for detection of anti-CII IgG2b and IgG1, using a standard in arbitrary U/ml; standard = 1,000 U/ml. **(c) **Delayed type hypersensitivity reactivity against CII. Ten mice were immunized with CII/complete Freund's adjuvant and implanted with osmotic pumps containing AMD3100 or PBS on day 7. On day 17 after immunization, five mice in each group were challenged with 10 μg of CII in the right ear and vehicle in the left. Delayed type hypersensitivity responses were measured as the percentage of ear swelling (i.e. 100 × the difference between the increase of thickness of the right and the left ear, divided by the thickness of the ear before challenge) at the indicated times. Bars represent averages ± standard error of the mean for five mice.

Total anti-CII IgG was determined in sera of the mice that were sacrificed for histological analysis. Titers of these antibodies in AMD3100-treated mice were not different from those in PBS-treated mice (Fig. 2a). The remainder of the sera were pooled and analysed for IgG2a, IgG2b and IgG1 isotypes against CII. IgG2a was below detection limit in both groups. We found no difference in the IgG2b and IgG1 concentrations between the two groups (Fig. 2b). Thus, the absence of clinical and histological symptoms of arthritis in the AMD3100-treated mice appeared not to be associated with any decreased antibody response against CII, nor with a switch between isotypes.

Cellular-immune responsiveness to CII was tested in mice that were immunized with CII/CFA and that were implanted on day 7 with osmotic minipumps containing AMD3100 or PBS. DTH testing was done on day 17 after immunization (i.e., day 10 of the treatment) by injecting 10 μg of CII in the right, and vehicle (PBS) in the left ear. Bars in Fig. 2c represent the percentages of swelling of the CII-challenged ears, normalized to the swelling of the PBS-challenged ears. No inhibition of the DTH response to CII was observed in the AMD3100-treated group indicating that AMD3100 did not interfere with the cellular immune response to CII.

### AMD3100 blocks CXCL12-elicited cell migration *in vivo *and chemotaxis *in vitro*

To see whether AMD3100 inhibits CIA by blocking CXCL12-mediated tissue infiltration, we immunized a set of 16 mice with CII/CFA. At the time of disease onset (day 27) they were divided into two subgroups matched by average incidence and clinical score. One group was implanted with osmotic minipumps delivering AMD3100. In the control group, pumps were filled with PBS. An air pouch assay was done on day 14 after minipump implantation (day 41). In both the AMD3100-treated and the PBS-treated group, four of the mice received an injection into the air pouch with CXCL12 (2 μg in 1 ml of 0.9% (w/v) NaCl), and four other mice received an injection with 0.9% NaCl. Two hours after this challenge, cells were washed out of the air pouch using 2 ml PBS containing 2% (v/v) FCS.

**Figure 3:**
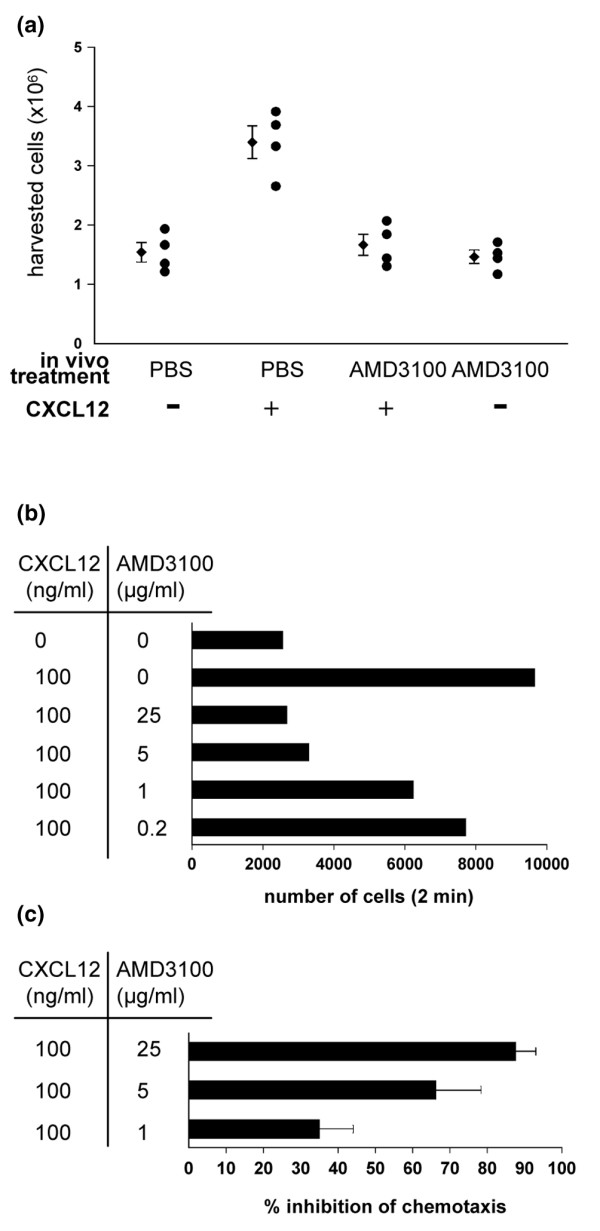
AMD3100 blocks CXCL12-elicited chemotaxis *in vivo *and *in vitro*. **(a) **Sixteen mice were immunized with collagen type II (CII) in complete Freund's adjuvant on day 0 and treated with AMD3100 or PBS in a similar way as described in the legend of Fig. 1. *In vivo *treatment is indicated along the X-axis. On the last day of treatment, a chemotactic assay was performed as described in Materials and methods. On that day, mice were injected with 2 μg of CXCL12 in 1 ml 0.9% NaCl (+) or 0.9% NaCl only (-) in a subcutaneous air pouch. Two hours after chemokine challenge, cells were washed out of the air pouch with 2 ml of PBS/FCS 2% and counted. Counts of the individual mice are shown (circles) and average ± standard error of the mean are indicated for each group (diamonds). **(b,c) **Dose-dependent inhibition by AMD3100 of CXCL12-elicited chemotaxis on total splenocytes. On day 21 post immunization with CII in complete Freund's adjuvant, spleens of three mice were pooled and a splenocyte suspension was prepared. Cell samples were pre-incubated for 10 minutes with AMD3100 at the indicated concentrations. Then, 5-μm filter inserts were loaded with 10^6 ^cells and transferred to a 24-well plate containing 100 ng/ml human CXCL12 in 600μl of buffer per well. After 3.5 h of incubation, the membrane inserts were removed and the cells in the wells were collected and counted by flow cytometry. The numbers of migrated cells of one representative experiment are shown in **(b)**. **(c) **The experiment was confirmed by three additional experiments and the data of the experiments were pooled and represented as the percentage inhibition ± standard error of the mean of CXCL12-elicited chemotaxis by the indicated concentrations of AMD3100.

Cell counts are shown in Fig. 3a. Mice implanted with PBS-delivering osmotic minipumps and challenged with 0.9% NaCl during the air pouch assay were considered as negative controls. In this group, an average of 1.5 ± 0.2 × 10^6 ^cells per mouse was obtained from the air pouch. Mice that carried PBS-delivering osmotic pumps and were injected with CXCL12 into the air pouch were considered as positive controls. In this group, we harvested an average of 3.4 ± 0.3 × 10^6 ^cells per mouse. This indicates specific infiltration of cells into the air pouch, in response to the chemokine CXCL12. Challenging mice with CXCL12 while they were treated with AMD3100 reduced the number of harvested cells to that of the negative control. The number of cells in the air pouch of AMD3100-treated mice after challenge with 0.9% NaCl was similar to that in the negative controls, indicating that the AMD3100-treatment did not, as such, affect the number of cells in the air pouch. Furthermore, flow cytometric analysis of the spleen and the lymph nodes did not reveal effects of AMD3100 on the number or proportions of CD4^+^, CD8^+^, CD19^+ ^and CD11b^+ ^cells. Together these data led us to conclude that treatment with AMD3100 is able to block CXCL12-elicited infiltration *in vivo *so as to prevent infiltration into inflamed tissues.

*In vitro *chemotactic assays performed on splenocytes of immunized mice allowed us to investigate the dose-dependent inhibition of CXCL12-elicited chemotaxis by AMD3100 (Fig. 3b). The percentage of cells that migrated in response to CXCL12 gradually decreased when the cells were pre-incubated with increasing concentrations of AMD3100. The dose-dependent inhibition by AMD3100 was confirmed in three additional experiments (pooled data are represented in Fig. 3c as the mean percentage of inhibition of CXCL12-elicited chemotaxis).

Flow cytometric analysis was performed after chemotaxis and revealed that CD4^+^, CD8^+^, CD19^+ ^and CD11b^+ ^cells were all attracted to CXCL12 with a chemotactic index of 2.5, 2.7, 6.9 and 3.4, respectively.

### Expression of CXCL12 and presence of CXCR4^+ ^cells in the arthritic joint

**Figure 4:**
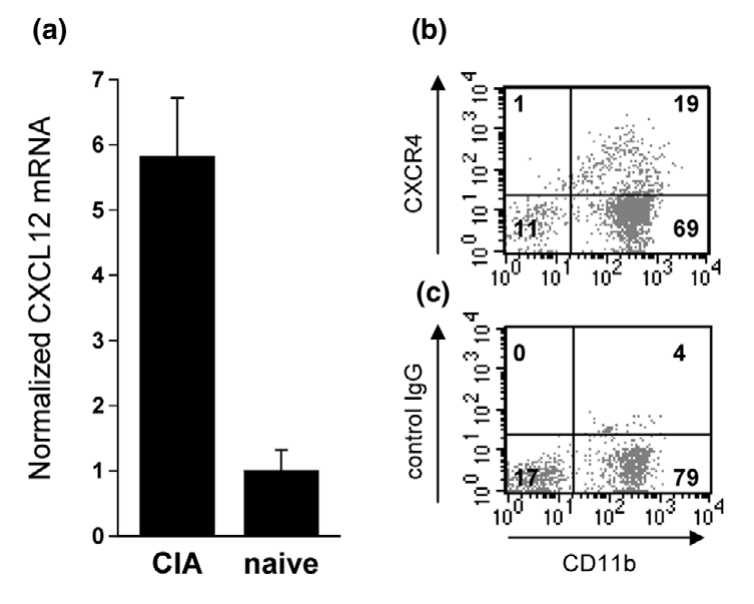
Presence of CXCL12 RNA and CXCR4^+ ^cells in the arthritic joint. **(a) **Synovia of three collagen type II/complete Freund's adjuvant-immunized collagen-induced arthritic (CIA) mice and three naive mice were isolated on day 35 after immunization and total RNA was purified. Reverse-transcription was performed and cDNA was subjected to quantitative PCR and normalized to the amount of 18S RNA. **(b) **Joints of three other collagen type II/complete Freund's adjuvant-immunized mice were washed at day 35 with PBS/FCS 2%. Cells that were harvested from the joint were stained for the presence of CD11b using fluorescein isothiocyanate (FITC)-labeled antibodies, and for CXCR4 using phycoerythrin (PE)-labeled antibodies. **(c) **Control staining for CXCR4 using a PE-labeled rat IgG2b isotype control antibody. One representative experiment out of two is shown.

To collect further evidence for the hypothesis that AMD3100 protects mice from arthritis by blocking CXCL12-mediated leukocyte mobilization, we ascertained that CXCL12-elicited migration of immunocompetent cells to inflamed sites does take place during CIA development. Numbers of CXCL12 mRNA copies were found to be elevated in synovial cells of the inflamed joint, as evident from quantitative reverse transcription (RT)-PCR (Fig. 4a). Among cells harvested by synovial lavage from the arthritic joint, an average of 15% stained double positive for CXCR4 and CD11b, as investigated by flow cytometry (Fig. 4b). These data were confirmed in an additional experiment. Taken together, these findings are indicative of CXCL12-elicited recruitment of CXCR4^+^CD11b^+ ^leukocytes to the joints as a mechanism contributing to CIA pathogenesis.

### Influence of AMD3100 on cytokine production

**Figure 5:**
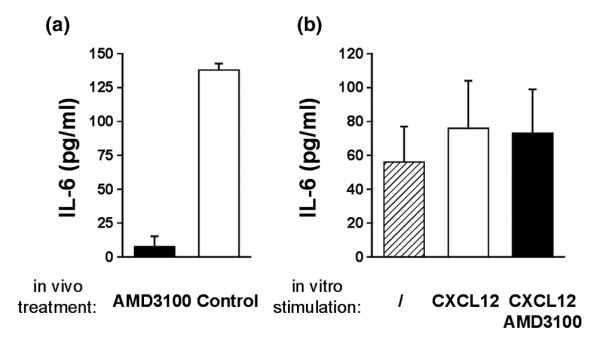
IL-6 levels in serum and in CXCL12-stimulated splenocyte cultures. **(a) **Eight mice were immunized with collagen type II/complete Freund's adjuvant (CIA) and implanted with osmotic minipumps delivering AMD3100 (four mice) at a constant rate of 600 μg/day or containing PBS (four mice). Blood was collected at day 10 of the treatment. Sera were pooled in each group and analysed for the presence of IL-6 (using a SearchLight Proteome array). Bars represent the average ± standard error of the mean of two independent experiments. **(b) **Splenocytes of three collagen type II/complete Freund's adjuvant-immunized mice were pooled and cultured in the absence of CXCL12, in the presence of CXCL12 (0.5 μg/ml) or in the presence of CXCL12 and AMD3100 (25 μg/ml). Supernatant was analysed after 48 h for the presence of IL-6. Bars represent the average ± standard error of the mean of three independent experiments.

We also considered the possibility that, in the course of CIA pathogenesis, CXCL12 might stimulate or enhance production of certain cytokines and that this might be a pathway by which AMD3100 could exert its protective action. To test this possibility, we looked at possible differences in the cytokine profiles of PBS- and AMD3100-treated mice. Eight mice were immunized with CII/CFA and treated on day 25 with AMD3100 (four mice) or PBS (four mice), using the osmotic minipumps. On day 35 post immunization (day 10 of the treatment), mice were bled and serum levels of IL-1β, IL-2, IL-4, IL-6, IL-10, IL-12, TNF-α and IFN-γ were determined by SearchLight proteome array. Only IL-6, IL-10, IL-12 and IFN-γ were detectable in the sera of mice. AMD3100 failed to change the levels of IL-10, IL-12 and IFN-γ, although blood levels of IL-6 were decreased in AMD3100-treated mice, a finding that was confirmed in additional experiments (data from these experiments are shown in Fig. 5a). Decreased systemic production of IL-6 in AMD3100-treated mice may be an indirect effect of inhibition of CXCL12-mediated cell traffic, as this might reduce formation of inflammatory tissue in joints and possibly other sites in the CII/CFA-immunized mice. Alternatively, inhibited IL-6 production might signify that CXCL12, aside from its chemotactic activity, directly activates certain CII/CFA-exposed leukocytes to produce this cytokine. To help distinguish between these two possibilities, we tested the ability of CXCL12 to induce the production of IL-6 in splenocyte cultures. Splenocytes of CII/CFA-immunized mice were cultured in the absence or presence of CXCL12 (0.5 μg/ml), with or without AMD3100 (25 μg/ml) (Fig. 5b). IL-6 was detectable in the supernatants of unstimulated cultures. Stimulation with CXCL12 or CXCL12 + AMD3100 did not alter the IL-6 production in the cultures, suggesting that the decreased IL-6 blood concentrations in the AMD3100-treated arthritic mice reflected an indirect, rather than a direct, CXCL12 action on IL-6 production.

### CXCL12 facilitates osteoclast differentiation and activation

**Figure 6:**
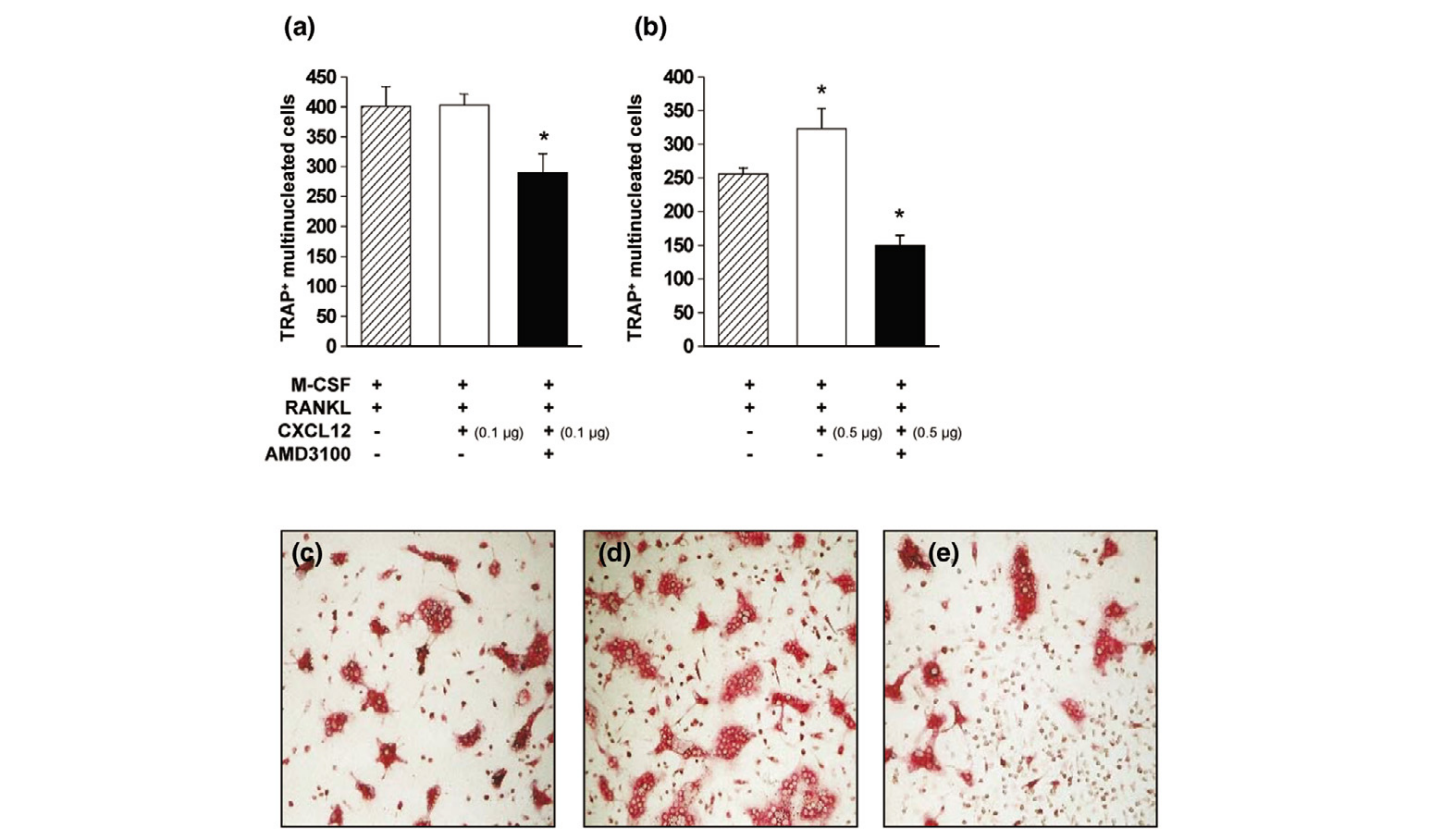
CXCL12 stimulates and AMD3100 inhibits osteoclast differentiation. Splenocytes of three collagen type II/complete Freund's adjuvant-immunized mice were isolated and pooled. **(a,b) **Splenocytes were cultured for 6 days in the cups of a chamberslide, in the presence of the indicated stimuli (macrophage colony-stimulating factor (M-CSF), 20 ng/ml; receptor activator of NF-κB ligand (RANKL), 100 ng/ml; CXCL12, 0.1 μg/ml in **(a)**, 0.5 μg/ml in **(b)**; AMD3100, 25 μg/ml). After stimulation, cells were fixed and stained for the presence of tartrate-resistant acid phosphatase (TRAP). TRAP^+ ^multinucleated (three or more nuclei) cells were counted within each cup. Bars represent averages ± standard error of the mean for four cultures. The asterisk represents p < 0.05 compared with the hatched bar (Mann-Whitney U-test). Representative pictures for TRAP-stained cultures stimulated with **(c) **M-CSF and RANKL alone and with added **(d) **CXCL12 (0.5 μg/ml) or **(e) **CXCL12 and AMD3100.

Osteoclast precursor cells and *in vitro *differentiated mature osteoclasts have been found to express CXCR4 [21-23], a finding that was confirmed in our laboratory (data not shown). To test the hypothesis that CXCL12 might facilitate osteoclast differentiation, splenocyte suspensions were cultured in the presence of M-CSF and RANKL, in the presence or absence of CXCL12 and/or AMD3100. After 6 days, osteoclasts were identified by staining for TRAP, a marker enzyme for osteoclasts. In cultures stimulated with M-CSF and RANKL, osteoclast differentiation could be observed (Fig. 6a–c). Addition of CXCL12 at a concentration of 0.1 μg/ml did not influence the number of osteoclasts (Fig. 6a). At 0.5 μg/ml, however, significantly higher numbers of osteoclasts were observed (Fig. 6b,d). Interestingly, when both AMD3100 and CXCL12 (in either concentration) were added, less differentiated osteoclasts appeared than in control cultures receiving only M-CSF and RANKL (Fig. 6a,b,e). Reduced differentiation of osteoclasts was not associated with increased mortality of splenocytes in these cultures (data not shown).

**Figure 7:**
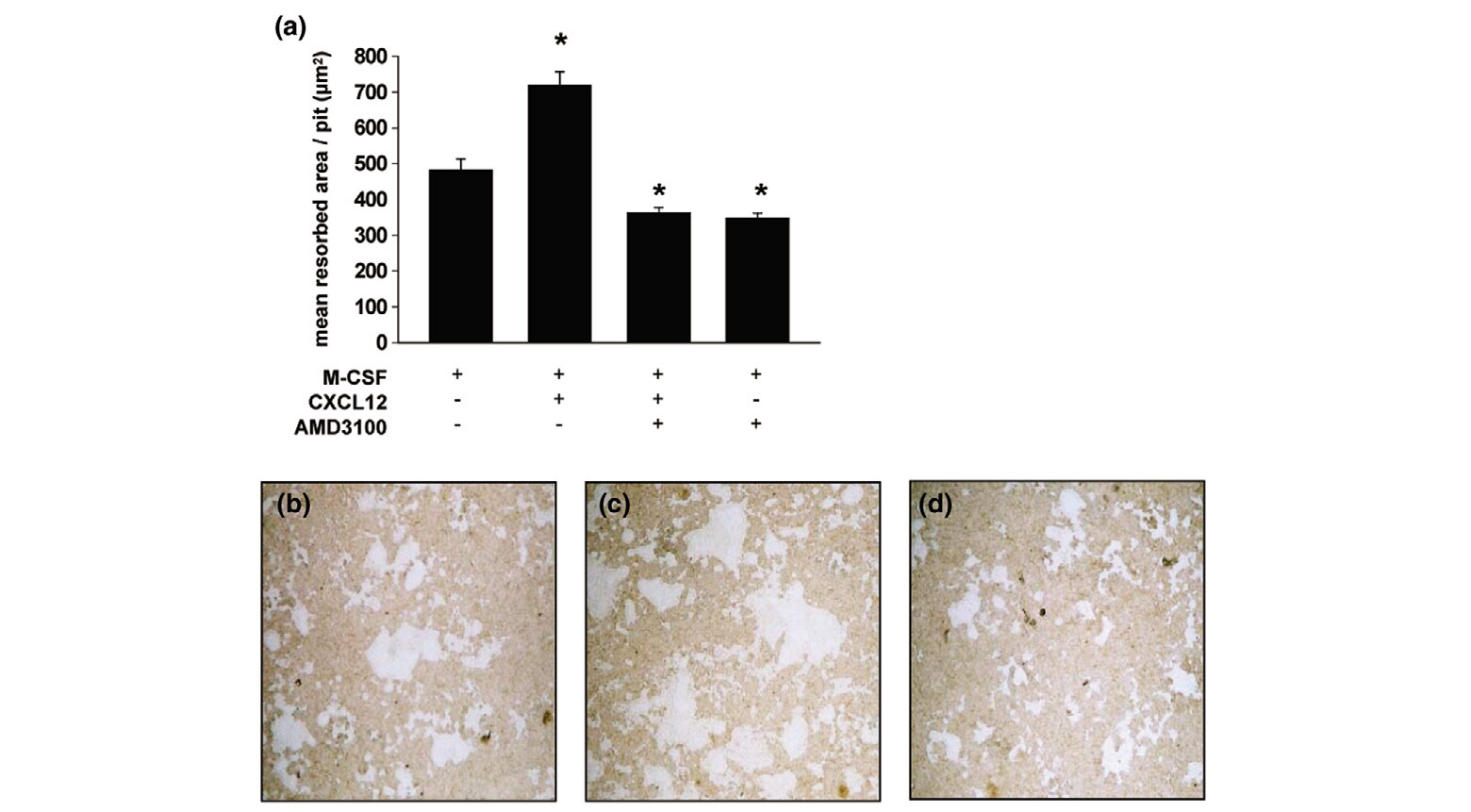
CXCL12 increases and AMD3100 inhibits osteoclast activity. Splenocytes of three collagen type II/complete Freund's adjuvant-immunized mice were isolated and pooled. Cell suspensions were cultured for 6 days on a quartz substrate coated with a calcium phosphate film in the presence of macrophage colony-stimulating factor (M-CSF, 20 ng/ml) and receptor activator of NF-κB ligand (RANKL, 100 ng/ml). At day 6 media were removed, cultures were provided with fresh media and stimulated as indicated (M-CSF, 20 ng/ml; CXCL12, 0.5 ng/ml; AMD3100, 25 μg/ml). Cells were removed from the quartz substrate after 2 days and resorption pits were visualized by light microscopy. The resorbed area was measured by a bioquant image analysis system. Bars represent the mean area resorbed by 1 osteoclast (average ± standard error of the mean), measured as the area of 1 resorption pit. The asterisk represents p < 0.001 compared with the M-CSF condition (Mann-Whitney U-test). Representative pictures of resorption pits are shown for the condition stimulated with **(b) **M-CSF, **(c) **M-CSF + CXCL12 and **(d) **M-CSF + CXCL12 + AMD3100. The data in this figure are representative for two independent experiments.

We also tested the effect of CXCL12 on the osteoclasts' ability to dissolve bone mineral. Splenocytes were cultured on quartz substrates, coated with a calcium phosphate film. Cells were stimulated with M-CSF + RANKL. After 6 days, multinucleated giant cells could be seen by microscopical examination, but resorption of the calcium phosphate film was not yet visible. On that day, the supernatant fluid was replaced with medium containing M-CSF alone, M-CSF + CXCL12 (0.5 μg/ml), M-CSF + CXCL12 + AMD3100 or M-CSF + AMD3100. Two days later, osteoclast activity was quantified as the ability to resorb the calcium phosphate film; 1 resorption pit is the area resorbed by 1 osteoclast, and the area of the pit correlates with osteoclast activity. The mean area of the resorption pits in the different conditions was calculated using a bioquant image analysis system (the data are presented in Fig. 7a and representative pictures of the resorbed areas on the calcium phosphate film are shown in Fig. 7b–d). It can be seen that CXCL12 significantly increased osteoclast activity, as evident from an increase in the resorbed area. When AMD3100 was added to the cultures with or without CXCL12, the osteoclast activity decreased significantly to a level beneath that of cultures with M-CSF alone (Fig. 7a). When the number of pits were counted and grouped according to their size, it appeared that CXCL12 also increased the number of pits, irrespective of their size, although resorption pits with a large area (>5,000 μm^2^) were most affected by CXCL12. In contrast, such large pits were barely detectable in cultures that had been treated with AMD3100 (Table 2).

Because AMD3100 decreased osteoclast differentiation (Fig. 6) and activation (Fig. 7) to a level beneath that of cultures where no exogenous CXCL12 was added, we verified whether splenocytes spontaneously produced CXCL12. To this end, splenocytes were cultured for 2 days without stimulation and CXCL12 concentrations in the supernatant were determined using the SearchLight proteome array. The mean level of CXCL12 in the supernatant of three independent splenocyte cultures was 85 ± 26 pg/ml.

Taken together, these data reveal a positive effect of CXCL12 on osteoclast differentiation and activity. Moreover, the inhibition of osteoclast differentiation and activation by the CXCR4 antagonist suggests an important role for endogenous CXCL12 in both processes.

## Discussion

We had already established that CXCL12 plays an important role in the pathogenesis of murine CIA in the highly sensitive IFN-γR KO mouse [13]. In particular, treatment with the specific CXCL12 inhibitor AMD3100 had been shown to afford protection against CIA. Examination of the underlying mechanism led to the conclusion that AMD3100 interfered with CXCL12-mediated immigration of leukocytes in the joints, but also reduced the systemic DTH against CII that, in IFN-γR KO mice, is typically more pronounced than in wild-type mice.

Here, we demonstrate that AMD3100 reduced the incidence and progression of CIA in IFN-γR-competent mice, in a similar manner to that previously demonstrated in IFN-γR KO mice [13]. Thus, irrespective of whether the IFN-γ system is defective or intact, CXCL12 is a key cytokine in the pathogenesis of murine CIA.

Quantitative RT-PCR revealed an increased presence of CXCL12 mRNA in the inflamed synovium in comparison with normal synovium, and 15% of the cells that could be harvested from inflamed joints were found to be CD11b^+^CXCR4^+ ^double positive. Splenocytes from mice subjected to the CIA immunisation schedule were found to display *in vitro *chemotactic responsiveness to CXCL12, an activity that was blocked by adding AMD3100. The *in vivo *relevance of these effects in mice immunized to develop CIA was examined with a subcutaneous air pouch system. CXCL12 injected into air pouches elicited immigration of leukocytes, an effect that was similarly blocked by AMD3100. These observations make it seem likely that in wild-type mice, as in IFN-γR-deficient ones, effects on leukocyte traffic constitute an important mechanism by which CXCL12 favours the pathogenesis of CIA.

In the case of IFN-γR KO mice, protection by AMD3100 treatment was associated with reduced cellular immune responsiveness to CII, as evident from reduced DTH in footpad swelling tests [13]. In wild-type mice, in contrast, AMD3100 did not affect DTH reactivity against CII. Basal DTH to CII following CIA induction was less pronounced in wild-type than in IFN-γR KO mice, however, and this in itself might account for it not being further reduced by AMD3100. Of note, another CXCL12-CXCR4 inhibitor, 4F-benzoyl-TN14003, has been shown to inhibit DTH to sheep red blood cells in normal Balb/c mice [24]. The difference in mouse strain and the use of a different antigen may account for the discrepancy between our findings. Failure of AMD3100 to affect anti-CII DTH reactivity in our wild-type mice suggests that, under the circumstances, cellular immunity to CII is perhaps not a key element by which CXCL12 influences the pathogenesis of CIA.

Formation of antibodies to CII was similarly not affected by AMD3100 treatment. Thus, although CXCL12 is known to act as a B cell growth factor [25], its possible action on humoral immunity to CII cannot be considered as a mechanism by which it acts as a disease-promoting factor in wild-type DBA/1 mice.

We also considered the possibility that CXCL12 favours CIA development by somehow affecting systemic cytokine production. In wild-type DBA/1 mice immunized to develop CIA, we found production of circulating IL-6, and levels of this cytokine, were reduced in mice treated with AMD3100. IL-6 is a crucial cytokine for CIA development because treatment with antibodies against IL-6 inhibits disease development [15]. In RA patients, serum IL-6 concentrations correlate with disease activity and decrease after effective treatment with disease modifying antirheumatic drugs. We wondered whether CXCL12 could induce IL-6 production and if this could be inhibited by AMD3100. If so, this would be an additional mechanism for AMD3100 to inhibit CIA development. We found, however, that IL-6 production was not increased in CXCL12-stimulated splenocyte cultures compared to unstimulated ones. This suggests that the lower levels of IL-6 in the serum of AMD3100-treated mice probably reflects the effectiveness of the CIA treatment, occurring for example by inhibition of leukocyte infiltration in the joint.

Furthermore, we investigated the possibility that CXCL12 can induce production of RANKL and thereby stimulate differentiation and/or activation of osteoclasts. If real, these activities might constitute part of the role of CXCL12 in CIA pathogenesis and might in part explain the protective effect of AMD3100. In fact, we found CXCL12 to be unable to induce RANKL or RANK expression and osteoclast differentiation in plain splenocyte cultures. The chemokine did potentiate induction of osteoclasts in cultures exposed to RANKL plus M-CSF, however, although it should be noted that the chemokine dose required to see this effect was in large excess of levels normally seen in CXCL12 production systems. Addition of AMD3100 to the system annihilated the CXCL12 effect but, intriguingly, reduced osteoclast induction to a level lower than that seen in cultures not exposed to CXCL12. A possible explanation may be that cultures exposed to M-CSF plus RANKL release endogenous CXCL12 at a level such that osteoclast induction is near to maximal, requiring supra high doses of exogenous CXCL12 for further augmentation.

Grassi *et al. *[26] reported that CXCL12 can enhance bone resorbing activity of osteoclasts. We similarly found that stimulation of osteoclasts with CXCL12 augmented their calcium phosphate-resorbing capacity. Moreover, addition of AMD3100 to osteoclast cultures reduced their resorbing potential. This inhibitory effect took place even if no exogenous CXCL12 had been added, showing that the osteoclast-activating activity of CXCL12 operates at concentrations within the endogenous physiological range.

## Conclusion

As evident from our observations, we demonstrate that CXCL12 plays a crucial role in the CIA pathogenesis of fully IFN-γR-competent mice, as it was proven to be before in IFN-γR KO mice. The underlying mechanisms are diverse, however, and their relative impact may differ depending on whether the IFN-γ system is defective or intact. In both cases, effects on leukocyte migration to the inflamed joints seem to play an important role. An enhancing effect of CXCL12 on cellular immunity may play an additional important role in IFN-γR KO mice, which are considerably more sensitive to the disease; this mechanism seems to be of less importance in wild-type mice. Furthermore, we were able to document a potentiating effect of CXCL12 on osteoclast differentiation and activation, both of which were counteracted by AMD3100. These observations hold further promise for potential treatment of RA patients with CXCR4 antagonists.

## Abbreviations

BSA = bovine serum albumin; CFA = complete Freund's adjuvant; CIA = collagen-induced arthritis; CII = collagen type II; DTH = delayed type hypersensitivity; ELISA = enzyme-linked immunosorbent assay; FCS = fetal calf serum; IFN = interferon; IFN-γR KO = IFN-γ receptor knock-out; IL = interleukin; M-CSF = macrophage colony-stimulating factor; PBS = phosphate-buffered saline; PCR = polymerase chain reaction; RA = rheumatoid arthritis; RANK = receptor activator of NF-κB; RANKL = receptor activator of NF-κB ligand; RT-PCR = reverse transcription polymerase chain reaction; TRAP = tartrate-resistant acid phosphatase.

## Competing interests

The authors declare that they have no competing interests

## Authors' contributions

CIA induction and the disease evaluation were done by BDK and YM. KV implanted the minipumps. PM performed the histological evaluations. Quantification of humoral and cellular response was done by BDK. *In vitro *and *in vivo *chemotactic assays were performed by SH and BDK, respectively. PCR and flow cytometry were done by HK. LG and BDK did the *in vitro *experiments for cytokine detection, osteoclast differentiation and the pit-forming assays. BDK, PM, SH and DS designed the study. BDK, PM and AB prepared the manuscript. All authors participated in the interpretation of the data.

## Figures and Tables

**Table 1:** Inhibition of the incidence and mean score of CIA by treatment with AMD3100

Experiment number	Treatment^a^	Cumulative incidence (%)		Score of arthritis (mean ± SEM)	
		Start of treatment^b^	End of treatment^c^	All mice^d^	Arthritic mice only^e^

1	AMD3100	2/10 (20%)	3/10 (30%)^f^	1.4 ± 0.9^f^	4.7 ± 2.4
	Control	2/12 (17%)	9/12 (75%)	5.2 ± 0.9	6.2 ± 0.6
2	AMD3100	1/7 (14%)	2/7 (29%)	0.9 ± 0.6^f^	3.0 ± 1.0
	Control	2/7 (29%)	5/7 (71%)	2.4 ± 0.8	3.4 ± 0.7
3	AMD3100	2/8 (25%)	2/8 (25%)^g^	0.6 ± 0.5^g^	2.5 ± 1.5
	Control	1/8 (13%)	7/8 (88%)	3.9 ± 1.4	4.3 ± 1.5

The table shows the results of three individual experiments. Male mice were immunized with collagen type II/complete Freund's adjuvant on day 0. ^a^At the day first symptoms appeared (day 27 in experiment 1 and 2, day 24 in experiment 3), mice were divided into two groups and were implanted with osmotic minipumps delivering AMD3100 at a constant rate of 600 μg/day or PBS in the control groups. Distribution of the mice between the two groups was done in a way that an equal incidence and a similar clinical score was reached in both groups. ^b^Arthritic incidence in both groups at the start of the treatment is shown. ^c^At the end of the treatment, there was a significant inhibition of the incidence in the AMD3100-treated group compared to the control in experiments 1 and 3 (^f^p < 0.05 and ^g^p < 0.01, respectively; binomial proportion test). ^d^At the end of the treatment, the mean arthritic scores calculated for all mice were significantly different between the AMD3100-treated and control groups for all the three experiments (^f^p < 0.05 for experiments 1 and 2; ^g^p < 0.01 for experiment 3; Mann-Whitney U-test). ^e^At the end of the treatment, the mean arthritic scores calculated for mice with symptoms of arthritis were not significantly different between the AMD3100-treated and control groups. SEM, standard error of the mean.

**Table 2:** CXCL12 increases and AMD3100 inhibits osteoclast activity

	Number of pits with indicated resorption area^a^				
*In vitro *stimulation^b^	50–100 μm^2^	100–500 μm^2^	500–1,000 μm^2^	1,000–5,000 μm^2^	>5,000 μm^2^

Control	481	1,002	166	153	22
CXCL12	848	1,854	382	346	90
CXCL12 + AMD3100	598	1,143	193	164	5
AMD3100	580	1,008	171	140	6

^a^The table shows the number of osteoclast resorption pits for five different surface intervals. ^b^Splenocytes of collagen type II/complete Freund's adjuvant-immunized mice were cultured as described in the legend of Fig. 8, and the resorbed area was measured.
